# Immune-Mediated Damage Completes the Parabola: *Cryptococcus neoformans* Pathogenesis Can Reflect the Outcome of a Weak or Strong Immune Response

**DOI:** 10.1128/mBio.02063-17

**Published:** 2017-12-12

**Authors:** Liise-anne Pirofski, Arturo Casadevall

**Affiliations:** a Department of Medicine, Division of Infectious Diseases, Albert Einstein College of Medicine and Montefiore Medical Center, Bronx, New York City, New York, USA; b Department of Molecular Microbiology and Immunology, Johns Hopkins Bloomberg School of Public Health, Baltimore, Maryland, USA

**Keywords:** fungi, pathogenesis, virulence

## Abstract

Cryptococcosis occurs most frequently in immunocompromised individuals. This has led to the prevailing view that this disease is the result of weak immune responses that cannot control the fungus. However, increasingly, clinical and experimental studies have revealed that the host immune response can contribute to cryptococcal pathogenesis, including the recent study of L. M. Neal et al. (mBio 8:e01415-17, 2017, https://doi.org/10.1128/mBio.01415-17) that reports that CD4^+^ T cells mediate tissue damage in experimental murine cryptococcosis. This finding has fundamental implications for our understanding of the pathogenesis of cryptococcal disease; it helps explain why immunotherapy has been largely unsuccessful in treatment and provides insight into the paradoxical observation that HIV-associated cryptococcosis may have a better prognosis than cryptococcosis in those with no known immune impairment. The demonstration that host-mediated damage can drive cryptococcal disease provides proof of concept that the parabola put forth in the damage-response framework has the flexibility to depict complex and changing outcomes of host-microbe interaction.

## COMMENTARY

Cryptococcosis is usually observed in hosts with impaired immunity. Consequently, the agent causing cryptococcosis, *Cryptococcus neoformans*, is often considered pathogenic only when the immune system is unable to control its growth. This has led to its characterization as an “opportunistic pathogen” (1). In their article in *mBio*, L. M. Neal et al. (2) report that the CD4^+^ T cell-mediated response to *C. neoformans* is a major contributor to tissue damage in cryptococcal meningitis in mice even though it also mediates fungal clearance. This observation adds to increasing evidence that the host response can drive cryptococcal disease and highlights the ability of the damage-response framework (DRF) to guide our understanding of microbial pathogenesis. The DRF was first put forth in 1999 (3) to provide a theory of microbial pathogenesis that could incorporate the contributions of both host and microbe to host damage that stems from host-microbe interaction. Prior to the DRF, microbial pathogenesis was largely viewed as a singular outcome of either microbial factors or host factors. While such microbe- or host-centered views were able to explain the pathogenesis of certain infectious diseases, they could not explain others, especially those caused by microbes only rarely associated with disease. This shortcoming became glaring in the late 1970s and early 1980s as the HIV/AIDS pandemic led to the emergence of previously rare and unusual diseases, including cryptococcosis (4).

In the original formulation of the DRF, the outcome of host-microbe interaction with different microbes was depicted by six curves that plotted host damage as a function of the strength of the immune response. These curves, referred to as pathogen classes, were based on what was known at the time about the outcome of infection with given microbes. The rationale for the pathogen classes was underpinned by the tenet that host damage can stem from microbial factors, host factors, or both. Central to this tenet was the idea that host damage stemming from the immune response to a microbe can drive disease pathogenesis. At the time the DRF was proposed, the host inflammatory response was not generally viewed as a causal factor in the pathogenesis of infectious diseases. However, this has changed. The occurrence of severe acute respiratory syndrome (SARS) in young, previously well persons who presented with excessive pulmonary inflammation (5), reminiscent of influenza epidemics that struck young, robust persons (6), highlighted the role that host-mediated damage can play in viral disease pathogenesis. The ability of the DRF to account for host damage due to inflammation stemming from the immune response to certain microbes (3) highlighted its flexibility and capacity to incorporate new diseases and information.

Despite the ability of the DRF to classify most microbes into one of the original six pathogen classes, new knowledge from clinical and experimental studies led to the conclusion that some of the original classifications were incorrect. Again, lessons learned from the HIV/AIDS pandemic provided new insights into microbial pathogenesis, perhaps best exemplified by cryptococcosis. *C. neoformans* was first classified as a class 2 pathogen, the definition of which was “pathogens that cause damage either in hosts with weak immune responses or in the setting of normal immune responses” (3). This characterization was consistent with available knowledge in 1999. However, the emergence of *Cryptococcus gattii* in apparently healthy persons in the Pacific Northwest (7) and the unexpected appearance of immune reconstitution inflammatory syndrome (IRIS)-associated cryptococcosis in patients with HIV/AIDS after initiation of anti-retroviral therapy (ART) (8, 9) revealed that the host immune response itself can contribute to the pathogenesis of cryptococcosis. Thus, classification of *C. neoformans* as a class 2 pathogen needed to be revisited.

**FIG 1:**
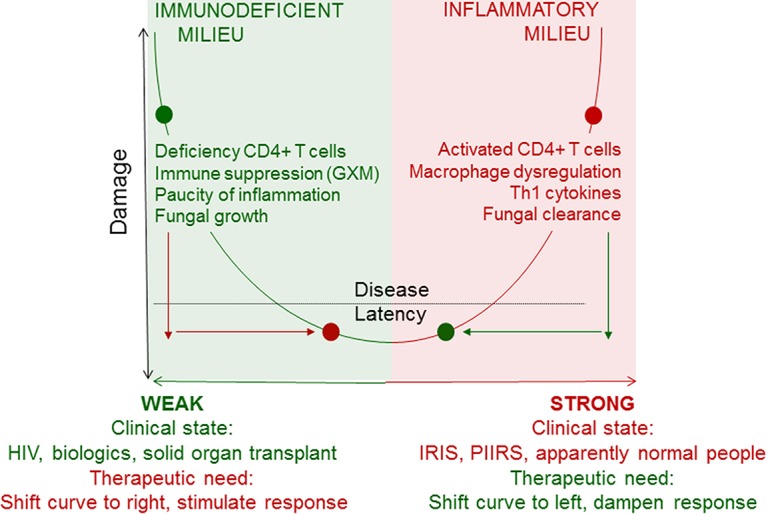
Outcomes of host-*Cryptococcus neoformans* interaction depicted by the basic parabola of the damage-response framework. The left side of the parabola, shaded in green and labeled weak, depicts the original circa 1999 concept that *C. neoformans* was a class 2 pathogen that caused damage in the setting of a weak or normal immune response. Here, the immune response fails to limit fungal growth, which results in host damage. The right side of the parabola, shaded in red and labeled strong, depicts information that has emerged since 1999, which has revealed that *C. neoformans* can elicit strong immune responses that produce host damage stemming from inflammation. The portion of the parabola that rises above the black dotted line represents the threshold for clinical disease. The portion of the parabola that lies below the black dotted line represents the state of *C. neoformans* latency, which is not associated with clinically evident host damage. Proposed therapeutic interventions, based on counteracting the main source of host damage are depicted by red (to enhance weak response) or green (to reduce strong response) arrows. Some factors that contribute to weak and strong immune responses are shown along the *y* axis for each type of response, though immunity is likely multifactorial, and additional factors are also likely to contribute (16, 29–35).

Another example of a pathogen needing reclassification is *Pneumocystis jirovecii*, which was originally classified as a class 1 pathogen, defined as “pathogens that cause damage only in the setting of weak immune responses” (3). However, pneumocystis pneumonia is associated with intense inflammation in children and after initiation of therapy in patients with HIV/AIDS (10, 11). In fact, a major advance in treating pneumocystis pneumonia was the realization that corticosteroid therapy reduced morbidity and mortality, a finding that was counterintuitive, since this disease arose in the setting of profound HIV-associated immunodeficiency. Thus, inflammation may drive pneumocystis- and cryptococcus-mediated damage, even in patients with severely impaired immunity, defying their initial classifications as class 1 and 2 pathogens, respectively. Although one might propose that these microbes be reclassified as class 3 pathogens, “pathogens that cause damage in the setting of appropriate immune responses and cause damage at both ends of the continuum of immune response” (3), most persons with normal immune responses never exhibit clinically detected damage as an outcome of interaction with these microbes. These examples highlight limitations of the original DRF pathogen classes and illustrate that each of the six classes is actually a derivative of a single parabola that can shift to the left, right, up, or down to depict the impact of the immune response on disease pathogenesis (Fig. 1).

The emergence of IRIS-associated cryptococcosis in the setting of ART initiation provided clear evidence that host damage in patients with cryptococcal disease may be driven by inflammation (12). IRIS often occurred in patients with very low initial cerebrospinal fluid (CSF) lymphocyte counts (8), and has been associated with chemokine dysregulation (13) and activated CD4^+^ T cells and macrophages (12, 14, 15). Similar findings have also been reported in some non-HIV-infected patients with cryptococcal meningitis (16, 17). A role for CD4^+^ T cells in the pathogenesis of cryptococcus-associated IRIS has also been demonstrated in experimental mouse models. Eschke and colleagues induced IRIS in RAG1^−/−^ mice by reconstituting them with naive (wild-type) CD4^+^ T cells 1 month after pulmonary infection with 500 CFU of *C. neoformans* (18). In this model, mice developed wasting and systemic inflammation evidenced by high levels of Th1-type cytokines (interleukin 6 [IL-6], tumor necrosis factor alpha [TNF-α], and gamma interferon [IFN-γ]), activated CD4^+^ T cells, and granulomatous inflammation in the liver compared to control mice that did not receive CD4^+^ T cells (and mice that received CD4^+^ T cells and were not infected). Reconstitution did not affect fungal clearance. This model demonstrated that CD4^+^ T cell reconstitution induced systemic inflammation in *C. neoformans*-infected mice that closely resembled ART-induced IRIS in HIV-infected patients with cryptococcosis.

Neal and colleagues (2) describe another mouse model in which CD4^+^ T cells mediate inflammation and host damage in the setting of *C. neoformans* infection. Their study was undertaken to gain insight into the role that CD4^+^ T cells may play in the pathogenesis of IRIS- and postinfectious inflammatory syndromes (PIIRS) in HIV-infected and HIV-uninfected patients with cryptococcosis. Mice infected intravenously with 10^6^ CFU of *C. neoformans* were monitored clinically or analyzed to determine their central nervous system (CNS) fungal burden (CFU), inflammatory pathology, and cellular and cytokine responses. The number of *C. neoformans* CFU in the brains of mice increased from 1 to 3 weeks after infection and then decreased from 3 to 5 weeks thereafter. Interestingly, the mice developed symptomatic disease and neurological deterioration 3 to 4 weeks after infection, corresponding to fungal clearance and surprisingly, mortality. CNS pathology revealed cellular inflammation marked by leukocytes beginning 3 weeks after infection that subsequently increased due to infiltration of CD4^+^ and CD8^+^ T cells, with CD4^+^ T cells being the predominant population and both populations being antigen experienced. Infiltrating CD4^+^ and CD8^+^ T cells exhibited a Th1-type bias, producing IFN-γ. Depletion of CD4^+^ T cells resulted in reduced mortality and inflammatory pathology, providing proof of concept that CD4^+^ T cells were the principal mediators of inflammation and damage in this model. Notably, survival of infected, CD4^+^ T cell-depleted mice was improved despite an inability to induce fungal clearance and markedly elevated fungal burdens compared to mice with sufficient CD4^+^ T cells.

The data generated by Neal et al. (2) demonstrate that CD4^+^ T cells can play dichotomous roles in *C. neoformans* infection. On one hand, they can mediate fungal clearance in the CNS, but on the other hand, they can induce inflammation that leads to neurological deterioration and death. This suggests a possible explanation for why inflammation is often a prominent feature of cryptococcal meningitis in HIV-uninfected, but not HIV-infected patients, and raises the intriguing possibility that the profound CD4^+^ T cell deficiency, which portends risk for cryptococcosis, may actually limit inflammatory pathology in HIV-infected persons not on ART. This dichotomy may also provide insight into the failure of adjunctive steroid therapy to reduce mortality or improve morbidity in HIV-associated cryptococcosis (19) and the fact that cryptococcosis may have a better prognosis in immunosuppressed patients than in nonimmunosuppressed patients (20–22). The detrimental effect of activated CD4^+^ T cells in mice also suggests the possibility that antifungal therapy-induced fungal clearance and reduced levels of glucuronoxylomannan (GXM) (an immune suppressing molecule [23–25]), could enhance CD4^+^ T cell activation and damage. This may help explain paradoxical worsening of cryptococcosis in certain patients after initiation of antifungal therapy (26).

The aforementioned experimental models provide possible mechanistic explanations for the dichotomous role that the immune response may play in the pathogenesis of IRIS- and PIIRS-associated cryptococcosis while contributing to fungal clearance. Thus, microbe and host both contribute to host damage and where an individual patient’s immune response lies on the continuum of the DRF parabola determines the nature of the disease process. At one end, on the left, a weak immune response can result in tissue damage due to fungal proliferation that results in compression of brain tissue with little or no inflammation in what is primarily microbe-mediated damage. At the other end, on the right, a strong cellular immune response can also damage brain tissue due to excessive inflammation in what is primarily host-mediated damage. The occurrence of host damage in the setting of weak and strong immune responses provides important clues as to why it has been so difficult to consistently use agents that modulate inflammation, such as corticosteroids and interferon, to improve the outcome of cryptococcosis. Although adjunctive IFN-γ therapy in HIV-associated cryptococcosis (27) and corticosteroids can ameliorate IRIS (28) in some patients, such immune-modulating agents may not be effective in other patients, because physicians do not usually know whether the preponderance of damage is due to host factors, microbial factors, or both. In this regard, the failure and detrimental effects of steroid therapy in patients with HIV-associated cryptococcosis (19) probably represent additional suppression of weak immunity resulting in enhancement of fungus-mediated damage. Hence, the effective use of immunotherapy is likely to require a rational approach that incorporates knowledge of where the immune response of an individual lies along the immune response co ntinuum depicted by the DRF parabola.
